# Primary tracheobronchial necrosis after esophagectomy: A nationwide multicenter retrospective study in Japan

**DOI:** 10.1002/ags3.12625

**Published:** 2022-10-08

**Authors:** Makoto Sakai, Hiroshi Saeki, Makoto Sohda, Mizuki Korematsu, Hiroshi Miyata, Daizo Murakami, Yoshifumi Baba, Ryo Ishii, Hiroshi Okamoto, Tomotaka Shibata, Ken Shirabe, Yasushi Toh, Akihiro Shiotani, The Japan Broncho‐Esophagological Society

**Affiliations:** ^1^ Division of Gastroenterological Surgery, Department of General Surgical Science, Graduate School of Medicine Gunma University Maebashi Japan; ^2^ Department of Head and Neck Surgery Osaka International Cancer Institute Osaka Japan; ^3^ Department of Digestive Surgery Osaka International Cancer Institute Osaka Japan; ^4^ Department of Otolaryngology‐Head and Neck Surgery, Graduate School of Medicine Kumamoto University Kumamoto Japan; ^5^ Department of Gastroenterological Surgery, Graduate School of Medical Science Kumamoto University Kumamoto Japan; ^6^ Department of Otolaryngology‐Head and Neck Surgery Tohoku University Graduate School of Medicine Sendai Japan; ^7^ Department of Gastroenterological Surgery Tohoku University Graduate School of Medicine Sendai Japan; ^8^ Advanced Trauma, Emergency and Critical Care Center/Gastroenterological and Pediatric Surgery Oita University Hospital Yufu Japan; ^9^ Department of General Surgical Science Gunma University Graduate School of Medicine Maebashi Japan; ^10^ National Hospital Organization Kyushu Cancer Center Fukuoka Japan; ^11^ Department of Otolaryngology‐ Head and Neck Surgery National Defense Medical College Saitama Japan

**Keywords:** esophageal neoplasms, esophagectomy, laryngeal neoplasms, pharyngeal neoplasms, tracheobronchial necrosis

## Abstract

**Background:**

The clinical features of postoperative primary tracheobronchial necrosis (P‐TBN; the necrosis without anastomotic leakage or other cervical and mediastinal abscess) remains unclear. This nationwide multicenter retrospective study first investigated the clinical features of P‐TBN after esophagectomy for upper aerodigestive tract cancer with a large cohort.

**Methods:**

As a study of the Japan Broncho‐Esophagological Society, a nationwide questionnaire survey was conducted in 67 institutions. The clinical data of 6370 patients who underwent esophagectomy for laryngeal, pharyngeal, and esophageal cancer between 2010 and 2019 were collected. Grades of P‐TBN were defined as follows: Grade 1, mucosal necrosis; Grade 2, transmural bronchial wall necrosis without fistula or perforation; Grade 3, transmural bronchial wall necrosis with fistula or perforation.

**Results:**

P‐TBN was observed in 48 (0.75%) of 6370 patients. The incidences of P‐TBN for pharyngo‐laryngo‐cervical esophagectomy (PLCE; n = 1650), total pharyngo‐laryngo‐esophagectomy (TPLE; n = 205), and subtotal esophagectomy (SE; n = 4515) were 2.0%, 5.4%, and 0.1%, respectively. The upper mediastinal LN dissection (*P* = 0.016) and the higher level of the tracheal resection (*P* = 0.039) were significantly associated with a higher grade of necrosis in PLCE and TPLE. Overall survival rates were significantly lower in patients with Grade 2 (*P* = 0.009) and Grade 3 (*P* = 0.004) than in those with Grade 1.

**Conclusion:**

The incidence of TBN restricted to P‐TBN was lower than previously reported. Maintaining the tracheal blood flow is essential to prevent worsening P‐TBN, especially in PLCE and TPLE. Our new P‐TBN severity grade may predict the outcome of patients with P‐TBN.

## Graphical abstract

The analyses of 6,370 patients revealed the incidence of primary tracheobronchial necrosis (P‐TBN) after esophagectomy is 0.75%. TBN severity associated with prognosis of patients with P‐TBN.

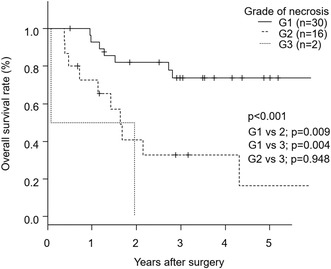

## 1 INTRODUCTION

Tracheobronchial necrosis (TBN) is a severe and potentially life‐threatening complication of esophagectomy for laryngeal, pharyngeal, and esophageal cancers, potentially causing subsequent tracheostomal stenosis, rupture of major vessels, and respiratory failure.^1^, ^2^ The prevalence of TBN was reported to be 0.2%–7.0% after esophagectomy^1^, ^3^ and 19.8% after pharyngo‐laryngo‐cervical esophagectomy (PLCE) and total pharyngo‐laryngo‐esophagectomy (TPLE).^2^

Tracheobronchial necrosis after esophagectomy is reported to have two different etiologies. Primary TBN (P‐TBN) is necrosis due to ischemic changes derived from the surgical procedures, while secondary TBN (S‐TBN) is due to anastomotic leakage or other cervical and mediastinal abscess.^4^ Few studies have investigated primary and secondary TBN separately.^1^, ^4^

Several factors are assumed to be associated with TBN. Inappropriate tracheal blood supply is one of the most critical factors.^1^ In PLCE and TPLE, lymph node dissection along trachea and creating tracheal stoma may reduce the tracheal blood supply dividing the tracheoesophageal artery, the lateral longitudinal anastomosis, and the connection between the thyroid gland and the tracheal wall. Devascularization of the trachea during mediastinal lymphadenectomy also has been reported to be associated with tracheobronchial lesions following esophagectomy.^1^, ^5^ The preservation of bronchial arteries (BA) is a widely accepted strategy to preserve respiratory function^6^ and to prevent ischemic changes of tracheobronchial lesions during esophagectomy.^7^ However, the right BA is often divided to improve esophageal mobilization during surgery.^8^, ^9^ Chemoradiotherapy (CRT) induces radiation injury in the irradiated field. Preoperative CRT was also reported to affect the bronchial blood flow^10^ and to be associated with tracheobronchial lesions after esophagectomy.^1^, ^3^ However, the association between those risk factors of tracheal blood supply reduction and P‐TBN has not been thoroughly investigated.

To investigate the clinical features of P‐TBN after esophagectomy, we conducted the first and largest nationwide multicenter retrospective study in Japan as a study of the Japan Broncho‐Esophagological Society (JBES). We herein explore the prevalence and effects of preoperative and procedural factors on P‐TBN. We also investigated the impact on the prognosis of P‐TBN using a new classification of the type and grade of the disease.

## 2 PATIENTS AND METHODS

### 2.1 Study design

The study was approved by the JBES (approval number 2020‐03) and the Ethics Committee of the Graduate School of Medicine, Gunma University (Protocol number HS2020‐132). Additionally, participating institutional Ethics Committees' approval was obtained as appropriate. Informed consent was obtained in the form of an opt‐out on the website. The inclusion criteria for registering patients through the survey questionnaire were: (a) pathologically diagnosed laryngeal, pharyngeal, and esophageal cancer; (b) underwent PLCE, TPLE, or subtotal esophagectomy (SE); (c) aged over 18 y; and (d) treated between January 2010 and December 2019. In this study, TBN was defined as necrosis of the cartilage or the pars membranacea of more than one tracheal ring.^2^ We restricted the cause of necrosis to ischemic changes derived from surgical procedures or preoperative therapy (primary necrosis). Cases of necrosis following anastomotic leakage or other mediastinal abscesses (secondary necrosis) and necrosis diagnosed before surgery were excluded.

### 2.2 Data collection

**FIGURE 1:**
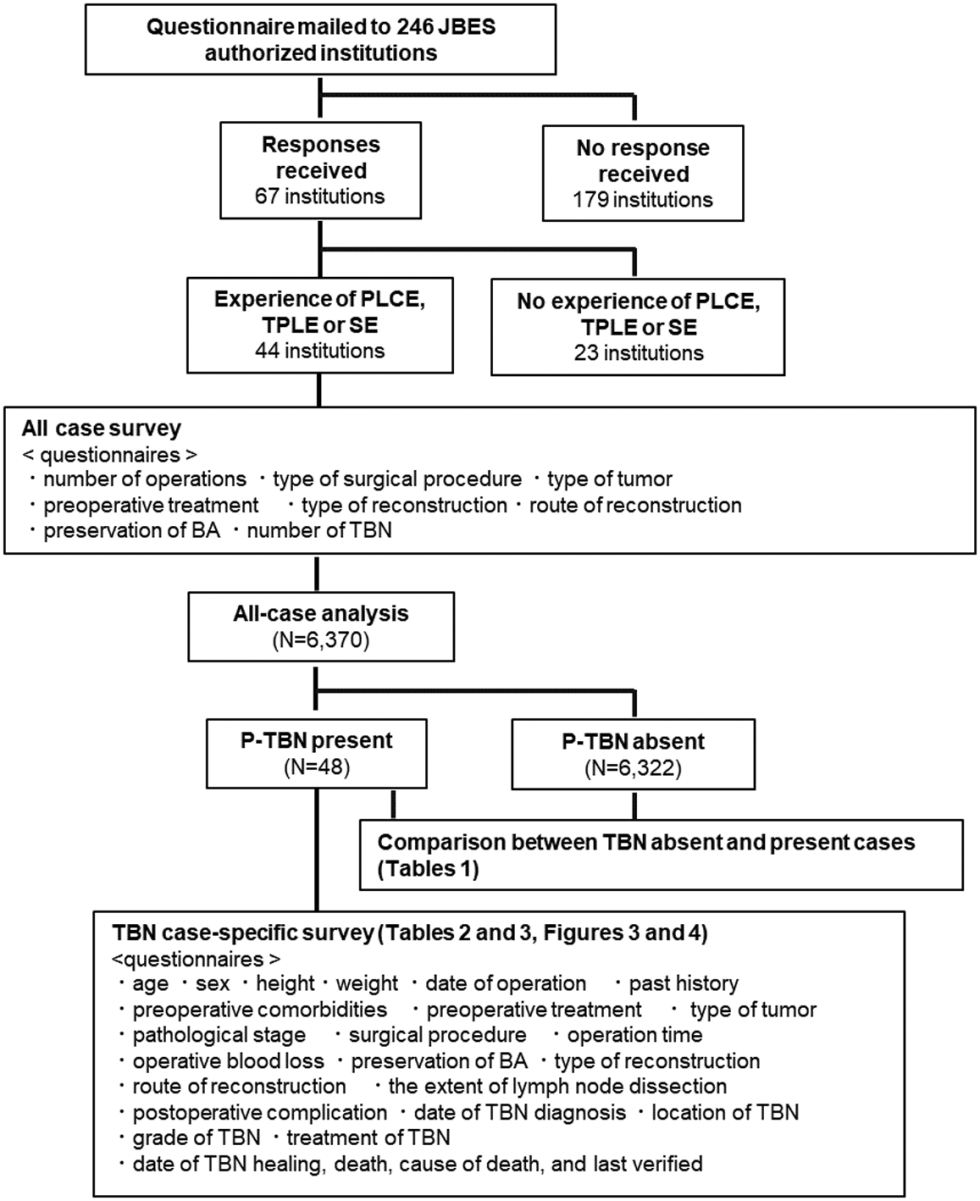
Flow chart of the survey. BA, bronchial arteries; PLCE, pharyngo‐laryngo‐cervical esophagectomy; SE, subtotal esophagectomy; TBN, tracheobronchial necrosis; TPLE, total pharyngo‐laryngo‐esophagectomy

Figure 1 shows a flow chart of the questionnaire survey. A questionnaire was sent by mail to 246 institutions authorized by the JBES as training institutions for board‐certified bronchoesophagologists. Sixty‐seven institutions returned the questionnaire forms. Of those, 23 institutions did not perform the specified operative procedures. Although our inclusion criteria restricted the type of tumor to laryngeal, pharyngeal, and esophageal cancer, 14 patients with different types of disease (two tracheal cancer, six thyroid cancer, one tracheal stenosis, five unknown causes) were included in the returned questionnaires. Considering that the surgical procedures of those cases matched the inclusion criteria, we included those 13 patients in the analysis. Ultimately, 6370 patients from 44 institutions were included in the study. The tumors were classified according to the 8th edition of the TNM classification of the International Union Against Cancer (UICC) AJCC.^11^ Postoperative complications were classified according to the Clavien–Dindo classification,^12^ and events classified as grade 3 or higher were documented as complications.

### 2.3 Classification of type and grade of TBN

**FIGURE 2:**
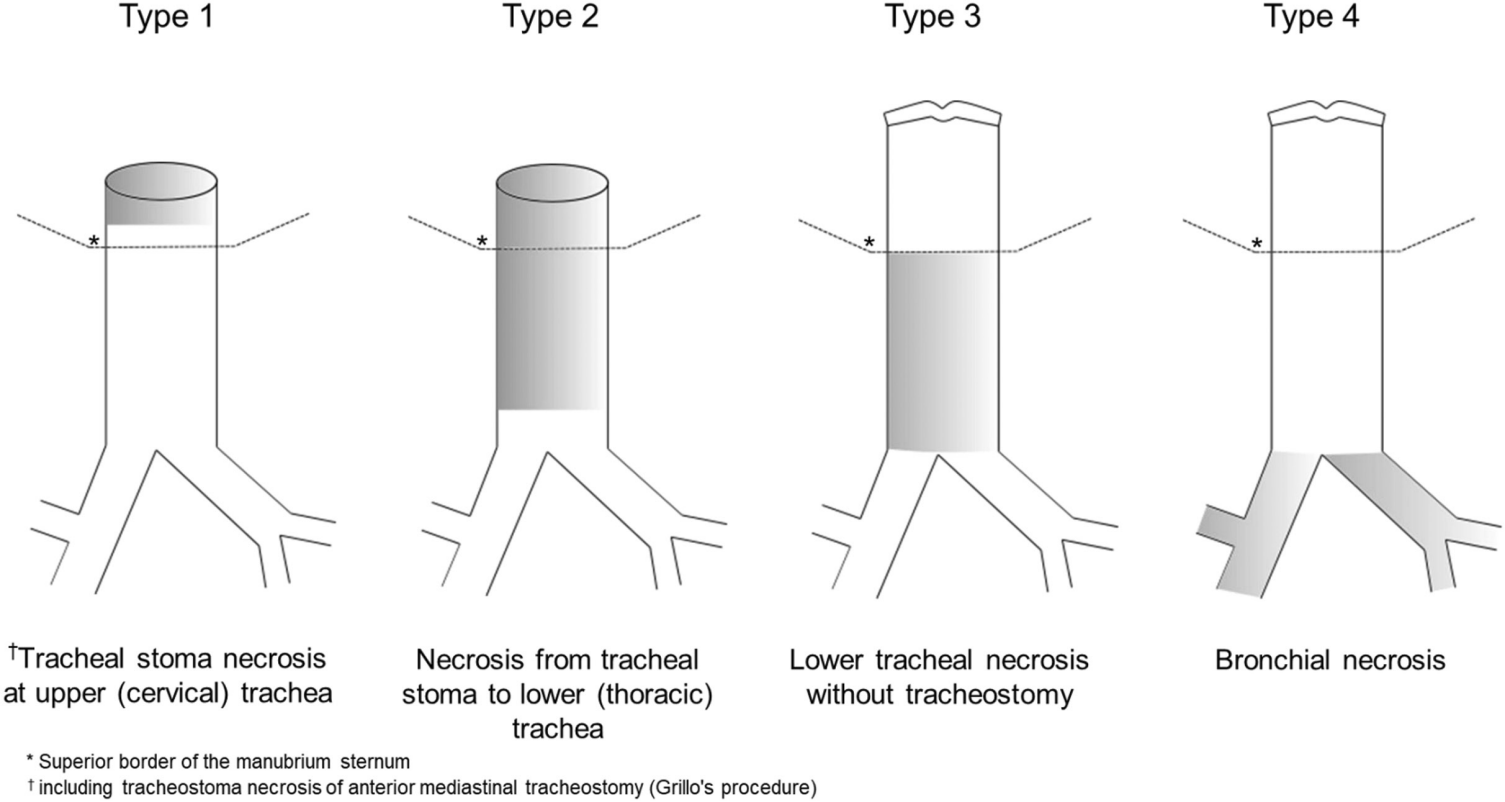
Type of P‐TBN according to the location and extent of necrosis

As shown in Figure 2, the type of TBN was defined depending on the location and extent of necrosis, as follows: (a) Type 1, tracheal stoma necrosis at the upper (cervical) trachea (including tracheostoma necrosis of anterior mediastinal tracheostomy [Grillo's procedure]); (b) Type 2, necrosis from the tracheal stoma to lower (thoracic) trachea; (c) Type 3, lower tracheal necrosis without tracheostomy; (d) Type 4, bronchial necrosis.

The grade of TBN was defined as follows: (a) Grade 1, mucosal necrosis (superficial fibrin adherence or dusky mucosa); (b) Grade 2, transmural bronchial wall necrosis without fistula or perforation (deep fibrin deposits with tracheal wall instability); (c) Grade 3, transmural bronchial wall necrosis with fistula or perforation. The TBN type, grade, and healing were confirmed by a combination of direct observation, bronchoscopy, and computed tomography (CT).

### 2.4 Statistical analysis

Subject characteristics were compared using the Mann–Whitney test, the Kruskal–Wallis test for categorical variables, and Fisher's exact test for continuous variables. The incidence of TBN and probabilities of TBN healing were estimated as a cumulative incidence and compared using Gray's test. The day of TBN healing was defined as the day on which TBN healing was achieved by conservative treatment or the day of successful surgical repair. Overall survival (OS) was calculated from the day of operation to the date of death or last follow‐up. P‐TBN‐specific survival was calculated from the day of operation to the date of P‐TBN‐related death or last follow‐up. Kaplan–Meier curves were generated for OS and P‐TBN‐specific survival, and significance was assessed using the log‐rank test. A probability value of <0.05 was considered significant. All statistical analyses were performed using EZR.^13^

## 3 RESULTS

### 3.1 Patient characteristics and incidence of P‐TBN among all cases

The baseline patient characteristics according to P‐TBN are summarized in Table 1. P‐TBN was observed in 48 (0.75%) of 6370 patients. The incidences of P‐TBN for PLCE, TPLE, and SE were 2.0% (33 of 1650 patients), 5.4% (11 of 205 patients), and 0.1% (4 of 4515 patients), respectively. The P‐TBN‐present group had hypopharyngeal cancer more frequently (41.7%) than the P‐TBN‐absent group (21.6%). The P‐TBN‐present group tended to have more PLCE and TPLE, and fewer SE, than the P‐TBN absent group (PLCE; 68.8 vs 26.0%, TPLE; 22.9 vs 3.1%, SE; 8.3 vs 71.0%). The P‐TBN‐present group had more frequent reconstruction using free jejunal grafts, reflecting the high rate of PLCE.

**TABLE 1:** Patient characteristics: All cases

Characteristics	All patients	P‐TBN		
		Absent	Present	
	(n = 6370)	(n = 6322)	(n = 48)	*P*‐value
Type of tumor (%)				
Laryngeal	87 (1.4)	85 (1.3)	2 (4.2)	<0.001
Hypopharyngeal	1387 (21.8)	1367 (21.6)	20 (41.7)	
Esophageal	4842 (76.0)	4822 (76.3)	20 (41.7)	
Multiple primary	40 (0.6)	34 (0.5)	6 (12.5)	
Others	14 (0.2)	14 (0.2)	0	
Preoperative treatment (%)				
None	3444 (54.1)	3423 (54.1)	22 (45.8)	<0.001
Chemotherapy	1946 (30.5)	1935 (30.6)	10 (20.8)	
CRT or RT	679 (10.7)	663 (10.5)	16 (33.3)	
Unknown	301 (4.7)	301 (4.8)	0	
Surgical procedure (%)				
PLCE	1650 (25.9)	1617 (26.0)	33 (68.8)	<0.001
TPLE	205 (3.2)	194 (3.1)	11 (22.9)	
SE	4515 (70.9)	4511 (71.0)	4 (8.3)	
Salvage surgery (%)				
Negative	5579 (87.6)	5546 (87.7)	33 (68.8)	<0.001
Positive	490 (7.7)	475 (7.5)	15 (31.2)	
Unknown	301 (4.7)	301 (4.8)	0	
Type of reconstruction (%)				
Whole stomach	24 (0.4)	22 (0.3)	2 (4.2)	<0.001
Gastric tube	4379 (68.7)	4369 (69.1)	10 (20.8)	
Jejunum (pedicled)	57 (0.9)	57 (0.9)	0	
Jejunum (free)	1576 (24.7)	1546 (24.5)	30 (62.5)	
Colon (pedicled)	155 (2.4)	155 (2.5)	0	
Colon (free)	8 (0.1)	8 (0.1)	0	
Skin flap	19 (0.3)	19 (0.3)	0	
Musculocutaneous flap	63 (1.0)	63 (1.0)	0	
Mixed	89 (1.4)	83 (1.3)	6 (12.5)	
Routes of reconstruction^a^ (%)				
Antethoracic (subcutaneous)	424 (9.0)	424 (9.0)	0	0.045
Retrosternal	1715 (36.3)	1713 (36.4)	2 (13.3)	
Posterior mediastinal	2251 (47.7)	2238 (47.6)	13 (86.7)	
Unknown	330 (7.0)	330 (7.0)	0	

Abbreviations: CRT, chemoradiotherapy; PLCE, pharyngo‐laryngo‐cervical esophagectomy; P‐TBN, primary tracheobronchial necrosis; RT, radiotherapy; SE, subtotal esophagectomy; TPLE, total pharyngo‐laryngo‐esophagectomy.

^a^ Analysis in patients undergoing TPLE and SE.

Preoperative treatment and salvage surgery were significantly different between the two groups (*P* < 0.001). The P‐TBN‐present group tended to have more frequent preoperative CRT, radiotherapy, and salvage surgery.

The association between BA preservation and incidence of P‐TBN, excluding 51 unknown cases, is shown in Table S1. The P‐TBN‐present group had more frequent BA preservation (*P* < 0.001), reflecting the higher incidence of P‐TBN in PLCE, in which no thoracic procedures are usually performed. When limited to TPLE and SE cases, the association between P‐TBN and BA preservation was not significant.

### 3.2 Patient characteristics and incidence of P‐TBN according to surgical procedure among all cases

The baseline patient characteristics according to P‐TBN in PLCE, TPLE, and SE are summarized in Tables S2‐S4. The P‐TBN‐present group in PLCE tended to have more frequent preoperative CRT, radiotherapy, and salvage surgery (*P* < 0.001) (Table S2). There were no significant differences in patient characteristics according to P‐TBN in patients who underwent TPLE and SE (Tables S3 and S4).

### 3.3 Association between patient characteristics and TBN grade in P‐TBN case‐specific survey

**FIGURE 3:**
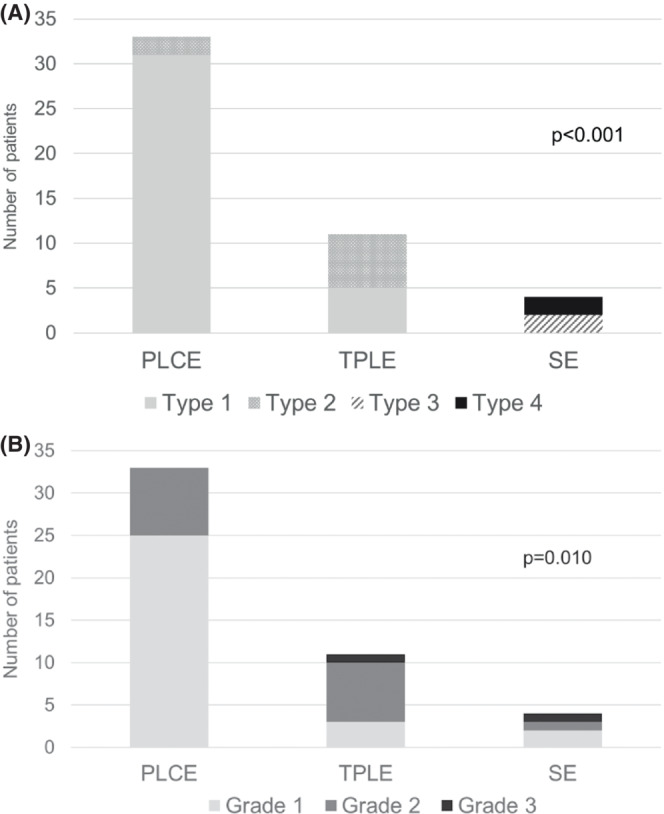
(A) Association between surgical procedures and type of P‐TBN. (B) Association between surgical procedures and grade of P‐TBN

The baseline patient characteristics with P‐TBN according to the grade of necrosis are summarized in Table 2. In patients with P‐TBN, the rates of Grade 1, 2, and 3 were 62.5%, 33.3%, and 4.2%, respectively. The type of reconstruction and reconstruction route significantly differed among TBN grades (*P* = 0.005 and 0.009). Grades 2 and 3 tended to have lower BA preservation rates (62.5% and 50.0%, respectively). PLCE and TPLE were associated with Type 1 and Type 2, reflecting the presence of tracheostomy. Type 3 and Type 4 were only seen in patients with SE (Figure 3A). The most common grades of necrosis in PLCE, TPLE, and SE were Grade 1 (75.8%), Grade 2 (63.6%), and Grade 1 (50.0%), respectively. Grade 3 was only observed in TPLE (n = 1, 9.1%) and SE (n = 1, 25.0%) (Figure 3B). The association between type and grade of TBN is shown in Figure S1.

**TABLE 2:** Characteristics of P‐TBN patients according to grade of necrosis

Characteristics	Grade of necrosis			
	Grade 1	Grade 2	Grade 3	*P*‐value
	(n = 30)	(n = 16)	(n = 2)	
Age, y (median [range])	70.0 [54.0, 84.0]	63.5 [50.0, 82.0]	67.0 [64.0, 70.0]	0.172
Sex (%)				
Female	8 (26.7)	2 (12.5)	0 (0.0)	0.660
Male	22 (73.3)	14 (87.5)	2 (100.0)	
BMI (median [range])	19.5 [15.3, 27.8]	19.3 [15.1, 27.0]	18.5 [16.0, 21.1]	0.809
Preoperative comorbidities (%)				
Negative	14 (46.7)	7 (43.8)	2 (100.0)	0.536
Positive	16 (53.3)	9 (56.2)	0 (0.0)	
Preoperative treatment (%)				
None	13 (43.3)	9 (56.2)	0	0.138
Chemotherapy	5 (16.7)	3 (18.8)	2 (100.0)	
CRT or RT	12 (40.0)	4 (25.0)	0	
Depth of tumor (%)				
T0‐1	2 (6.7)	3 (18.8)	0 (0.0)	0.459
T2‐4	28 (93.3)	13 (81.2)	2 (100.0)	
Lymph node metastasis (%)				
Negative	13 (43.3)	3 (18.8)	0 (0.0)	0.203
Positive	17 (56.7)	13 (81.2)	2 (100.0)	
Salvage surgery (%)				
Negative	19 (63.3)	12 (75.0)	2 (100.0)	0.561
Positive	11 (36.7)	4 (25.0)	0 (0.0)	
Operation time (min) (median [range])	663 [391, 922]	655 [464, 990]	683 [497, 869]	0.951
Blood loss (mL) (median [range])	450 [15, 1320]	627 [187, 996]	512 [70, 955]	0.254
Type of tumor (%)				
Laryngeal	2 (6.7)	0 (0.0)	0 (0.0)	0.189
Hypopharyngeal	15 (50.0)	6 (37.5)	0 (0.0)	
Esophageal	12 (40.0)	7 (43.8)	1 (50.0)	
Multiple primary	1 (3.3)	3 (18.8)	1 (50.0)	
Type of reconstruction (%)				
Whole stomach	1 (3.3)	0 (0.0)	1 (50.0)	0.005
Gastric tube	2 (6.7)	7 (43.8)	1 (50.0)	
Jejunum: Free	22 (73.3)	8 (50.0)	0 (0.0)	
Gastric tube + Jejunum (free)	2 (6.7)	1 (6.2)	0 (0.0)	
Jejunum (free) + flap	3 (10.0)	0 (0.0)	0 (0.0)	
Reconstruction routes (%)				
Cervical procedure only	25 (83.3)	8 (50.0)	0 (0.0)	0.009
Retrosternal	1 (3.3)	1 (6.2)	0 (0.0)	
Posterior mediastinal	4 (13.3)	7 (43.8)	2 (100.0)	
Lymph node dissection^a^ (%)				
Cervical	30 (100.0)	15 (93.8)	2 (100.0)	0.375
Upper mediastinal	6 (20.0)	9 (56.2)	1 (50.0)	0.023
Middle and lower mediastinal	3 (10.0)	5 (31.2)	1 (50.0)	0.096
Abdominal	2 (6.7)	4 (25.0)	1 (50.0)	0.063
Preservation of bronchial artery (%)				
Bilateral preserved	29 (96.7)	10 (62.5)	1 (50.0)	0.019
Lateral: Right divided	0 (0.0)	2 (12.5)	1 (50.0)	
Lateral: Left divided	0 (0.0)	1 (6.2)	0 (0.0)	
Bilateral divided	1 (3.3)	2 (12.5)	0 (0.0)	
Unknown	0 (0.0)	1 (6.2)	0 (0.0)	
Postoperative complications (CD ≥3) (%)				
Negative	25 (83.3)	12 (75.0)	1 (50.0)	0.356
Positive	5 (16.7)	4 (25.0)	1 (50.0)	
Treatment‐related factors				
Time to diagnosis, day (median [range])	6 [1, 80]	9 [3,25]	117 [26207]	0.030
Treatment (%)				
Conservative	28 (93.3)	5 (31.2)	2 (100.0)	<0.001
Surgical repair	2 (6.7)	11 (68.8)	0 (0.0)	
Time to healing, day (median [range])	13 [0, 948]	45 [7, 402]	NA^b^	0.055
Achieving of healing (%)	29 (96.7)	15 (93.8)	0 (0.0)	0.005
P‐TBN related death (%)	0 (0.0)	1 (6.2)	1 (50.0)	0.029

Abbreviations: CRT, chemoradiotherapy; Abbreviations: P‐TBN, Pprimary tracheobronchial necrosis; RT, radiotherapy.

^a^ Duplicated.

^b^ No patients with G3 necrosis archived healing.

In TPLE and PLCE cases, the upper mediastinal LN dissection and the higher level of the tracheal resection were significantly associated with a higher grade of necrosis (*P* = 0.016 and 0.039, respectively). However, thyroidectomy was not significant (Table 3).

**TABLE 3:** Association between upper mediastinal LN dissection, thyroidectomy, the number of resected trachea and grade of necrosis among patients undergoing PLCE and TPLE: P‐TBN cases

Characteristics	Grade of necrosis		
	Grade 1	Grade 2, 3	*P*‐value
	(n = 28)	(n = 16)	
Upper mediastinal LN dissection (%)^a^			
No	24 (75.0)	8 (25.0)	0.016
Yes	4 (33.3)	8 (66.7)	
Thyroidectomy (partial and total)^b^ (%)^a^			
No	10 (50.0)	10 (50.0)	0.075
Yes	10 (83.3)	2 (16.7)	
Level of tracheal resection^c^ (%)^a^			
Higher than the third tracheal ring	6 (42.9)	8 (57.1)	0.039
Lower than the third tracheal ring	18 (78.3)	5 (21.7)	

Abbreviations: PLCE, pharyngo‐laryngo‐cervical esophagectomy; P‐TBN, primary tracheobronchial necrosis; TPLE, total pharyngo‐laryngo‐esophagectomy.

^a^ ROW percentage.

^b^ Twelve unknown cases were excluded.

^c^ Eleven unknown cases were excluded.

### 3.4 Treatment‐related factors and prognosis of P‐TBN in TBN case‐specific survey

**FIGURE 4:**
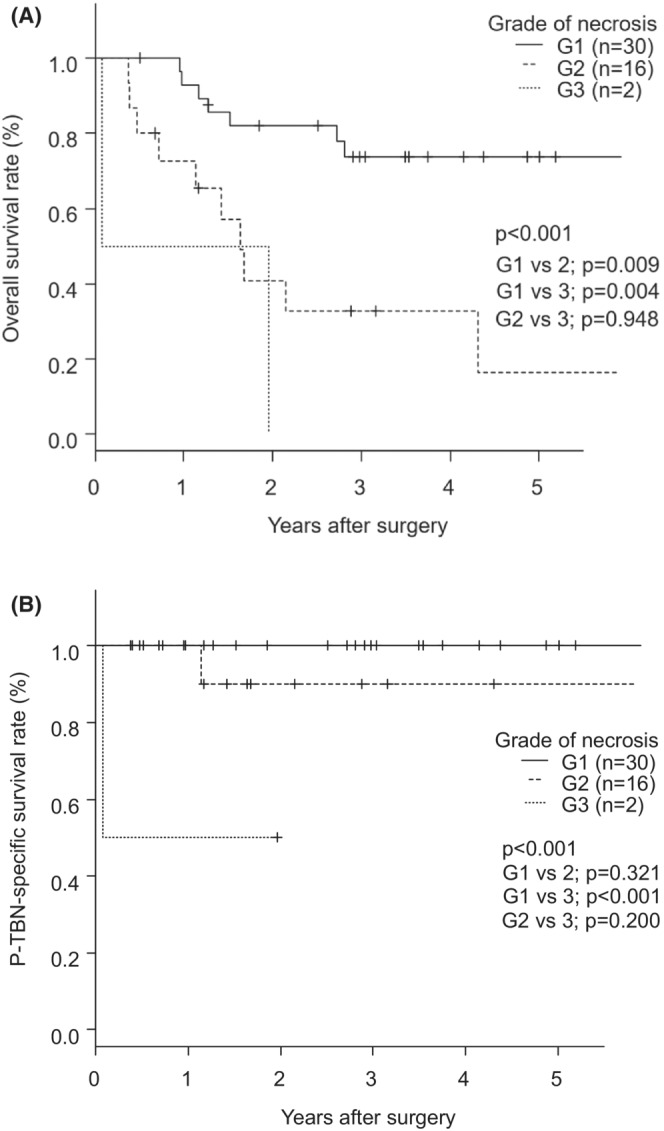
(A) Overall survival according to the grade of necrosis. (B) P‐TBN specific survival rate according to the grade of necrosis. [Correction added on 28 October 2022, after first online publication: Figure 4B has been corrected]

Treatment‐related factors are shown in Table 2. The median times to diagnosis of Grades 1, 2, and 3 were 6.0, 9.0, and 117 d, respectively (*P* = 0.030). Most cases of TBN occurred within 30 postoperative days, except for Grade 3 (Figures S2 and S3). Surgical repair was frequently performed in Grade 2 (*P* < 0.001). The OS rate was significantly higher for patients with Grade 1 than for those with Grades 2 or 3 (*P* < 0.001) (Figure 4A). Of 30 patients with Grade 1, most were hypopharyngeal cancer (n = 15) and cervical esophageal cancer (n = 10). Most hypopharyngeal cancer patients with Grade1 had stage III/IV disease (n = 10), and the 5‐y OS rate of those patients was 74.1%. The 5‐y OS of cervical esophageal cancer patients with Grade1 was 66.7%. The rates of achieving healing were 96.7% for Grade 1, 93.8% for Grade 2, and 0% for Grade 3 (*P* < 0.005). Two patients died from P‐TBN, accounting for an overall prevalence of P‐TBN‐related death of 0.03%. The P‐TBN‐specific survival rate was significantly lower for patients with Grade 3 than Grade 1 (*P* < 0.001) (Figure 4B). Both Grade 3 patients who died had trachea‐gastric tube fistula. One patient died from acute respiratory distress syndrome caused by fluid aspiration in the gastric conduit through the fistula on day 1 after the onset of TBN. The other patient died from lung metastasis on day 497 after the onset of TBN.

## 4 DISCUSSION

The most important findings of the present study were that the incidence of P‐TBN after esophagectomy was 0.75%, and TPLE had the most frequent incidence of P‐TBN (5.4%). The upper mediastinal LN dissection and the higher level of the tracheal resection were significantly associated with P‐TBN severity, defined by our new grading classification of TBN. P‐TBN severity was also associated with the survival of patients with P‐TBN after esophagectomy. To our knowledge, this is the first and largest nationwide multicenter retrospective study to investigate the clinical features and outcomes of P‐TBN after esophagectomy for upper aerodigestive tract cancer.

The prevalence of TBN was reported to be 0.2%–7.0% after esophagectomy^1^, ^3^ and 19.8% after PLCE and TPLE.^2^ The incidences of P‐TBN in our study for PLCE, TPLE, and SE were 2.0% (33 of 1650 patients), 5.4% (11 of 205 patients), and 0.1% (4 of 4515 patients), respectively. Our study revealed a lower incidence of TBN than previously reported if we restrict it to the P‐TBN. Primary and secondary necrosis have different etiologies.^4^ S‐TBN is caused by mediastinal inflammation, mainly following anastomotic leakage. Thus, S‐TBN could be recognized as a sequential complication of leakage or other mediastinal abscesses. Bartels et al^1^ retrospectively analyzed nonmalignant lesions of the trachea or main‐stem bronchi in 785 patients with esophageal cancer who underwent esophagectomy. They reported that the rate of tracheobronchial lesions from ischemia was 2.3%, whereas the rate of tracheobronchial lesions caused by peritracheal inflammation resulting from the anastomotic leak was 0.89%. Tanaka et al^4^ reported that the rate of P‐TBN was 6.1%, whereas the rate of S‐TBN was 4.0% in 49 patients who underwent salvage esophagectomy. Thus, the incidence of P‐TBN seemed to be 1.5–2.5 times that of S‐TBN. Our study indicated the need for the recognition that the two different etiologies underlie the overall prevalence of TBN.

Tracheobronchial blood supply was maintained by the bronchial artery and the tracheal sheath, including tracheoesophageal branches originating from the inferior thyroid artery, the subclavian artery, internal mammary artery, and innominate artery.^5^ Tracheostomy, LN dissection around the trachea, and thyroidectomy are often performed in PLCE and TPLE for laryngeal, pharyngeal, and cervical esophageal cancer. These procedures can potentially reduce the blood supply of the trachea by sacrificing the tracheoesophageal artery, the lateral longitudinal anastomosis, and the connection between the thyroid gland and the tracheal wall. Fujiki et al^2^ reported that the addition of total esophagectomy is the most important risk factor for tracheal necrosis after total pharyngolaryngectomy because most of the tracheoesophageal branches of the bronchial artery and the innominate‐subclavian system are sacrificed during esophageal dissection. In our study, TPLE had the most frequent incidence of P‐TBN (5.4%), and the upper mediastinal LN dissection and the higher level of tracheal resection were significantly associated with a higher grade of necrosis in PLCE and TPLE. Our data indicated the significance of maintaining the tracheal blood flow to prevent P‐TBN. When the LN dissection around the trachea was performed, avoiding the overlong remnant trachea is a possible preventable procedure for worsening P‐TBN.

Preoperative radiotherapy was also reported to affect the bronchial blood flow^10^ and to be associated with tracheobronchial lesions after esophagectomy.^1^, ^14^ Yamamoto et al investigated the bronchial mucosal blood flow in surgically‐resected lung cancer patients with a laser Doppler flowmeter. They revealed that the preoperative blood flow in patients with preoperative chemoradiotherapy decreased to 70% of those without preoperative therapy.^10^ Bartels et al^1^ reported that preoperative chemoradiotherapy for locally advanced tumors located at or above the level of the tracheal bifurcation was a significant independent predictive factor of postoperative tracheobronchial lesions. Tachimori et al^3^ analyzed 612 patients with esophageal cancer who underwent esophagectomy and reported a TBN incidence of 7% in patients with salvage esophagectomy, which was higher than that (0.2%) in patients with no preoperative therapy. In our study, the P‐TBN‐present group tended to have more frequent preoperative chemoradiotherapy and salvage surgery in PLCE. A history of radiation to the neck increases the risk of wound complications after head and neck surgery.^15^, ^16^ PLCE cannot prevent devascularization at the level of tracheostomy and tracheostomy; these areas are unavoidably located in the irradiated field when preoperative radiation is performed. These mechanisms may explain the association between preoperative chemoradiotherapy and a higher incidence of P‐TBN in PLCE. In contrast, preoperative chemoradiotherapy and salvage surgery were not associated with P‐TBN in TPLE and SE in our study. Our study could not assess the extent of devascularization around the trachea. However, the relatively widespread recognition among board‐certified bronchoesophagologists of the importance of preserving the tracheal blood supply in patients who undergo preoperative radiotherapy and salvage surgery may have introduced a procedure modification and affected our results. Several reports showed that a staged procedure is safe and effective for high‐risk patients, especially in TPLE.^17^, ^18^ Although our study had no data on whether the surgical procedure was staged or nonstaged, the staged approach might also be helpful for patients with the potential risk of tracheal blood supply reduction.

Preservation of BA is widely accepted to prevent respiratory dysfunction and ischemic damage of tracheobronchial lesions during esophagectomy.^6^, ^7^ However, whether or not the surgeon preserves the BA depends on the surgeon's choice or the hospital policy they belong to. Fujita et al^6^ reported that mediastinal lymph node dissection with preservation of BA and pulmonary branches of the vagus nerve helped to maintain postoperative respiratory function in radical esophagectomy. Nakahara et al investigated the intraoperative tracheal mucosal blood flow using a probe attached to the surface of the cuff of an endotracheal tube. They revealed that gentle clamping of the right BA significantly decreased tracheal mucosal blood flow.^7^ Although our findings showed no significant association between P‐TBN and BA preservation, the significant effect of the upper mediastinal procedure on P‐TBN severity implicates that BA preservation must be considered when severe reduction of upper tracheal blood flow is expected.

To the best of our knowledge, this is the first study using a new grading system for the severity of TBN. Our TBN grading system is associated with the rate of healing, P‐TBN‐related death, and poor survival of patients with P‐TBN after esophagectomy. Most cases of TBN Grade 1 were treated conservatively, whereas 70% of TBN Grade 2 cases underwent surgical repair. In contrast, patients with TBN Grade 3 could not achieve TBN healing. Chung et al^19^ reported that 5‐y OS of stage III/IV surgically treated hypopharyngeal cancer was 45.3%. Saeki et al^20^ reported that the 5‐y OS of surgically resected cervical esophageal cancer was 71.5%. In our study, the OS of stage III/IV hypopharyngeal and cervical esophageal cancer patients with Grade1 was not inferior to those previously described. It suggests that proper management for patients with Grade1 could achieve the same survival as patients without P‐TBN. Our graded classification of TBN severity will contribute to the precise assessment of TBN severity, selection of appropriate treatment strategies, and the prediction of outcome of patients with TBN.

This study had several limitations. First, this was a retrospective observational study in which surgeons at multiple institutions participated, and the answers to the questionnaire were retrospectively derived from medical records. Second, only specialized training institutions for board‐certified bronchoesophagologists were surveyed. Third, our study had unavoidable participation, nonresponse, and questionnaire bias. Fourth, our study did not have specific data about the radiation dose and field, which strongly affect the tracheal blood flow. The rationale for the applied surgical procedure, including the level of tracheostoma, the extent of lymph node dissection, and the accurate recognition of the importance of tracheal blood flow of each surgeon were also unknown based on the nature of the retrospective study. Fifth, we could not conduct a multivariate analysis to statistically identify the risk factors for P‐TBN due to our study design. A large‐scale prospective study could address these limitations but may be impractical or impossible because TBN is a rare complication. We believe that our results based on the large cohort notably contribute to providing novel and valuable information for this severe and rare complication.

In conclusion, the present multicenter retrospective study of 6370 patients revealed a P‐TBN incidence after esophagectomy of 0.75%, which was lower than the previously estimated TBN incidence. The incidence of P‐TBN for PLCE, TPLE, and SE were 2.0%, 5.4%, and 0.1%, repectively. Maintaining the tracheal blood flow is essential to prevent worsening P‐TBN, especially in PLCE and TPLE. Our TBN severity grading system predicts the outcome of patients with P‐TBN.

## DISCLOSURE

Conflict of Interest: Ken Shirabe is an editorial member of the *Annals of Gastroenterological Surgery*. The other authors declare that there are no conflicts of interest or funding associated with this article.

Author Contributions: M. Sakai and H.S. are the guarantors of the article. M. Sakai, HS, and M. Sohda were involved in the study design and data interpretation, and writing the first draft of the article. M.K., M.H., D.M., Y.B., R.I., H.O., and T.S. contributed substantially to data acquisition. M.K., M.H., D.M., Y.B., R.I., H.O., T.S., K.S., Y.T., and A.S. made critical revisions to the article and provided expert opinions on the implications of the study findings.

## ETHICAL APPROVAL

This retrospective study was approved by the JBES (approval number 2020‐03) and the Ethics Committee of the Graduate School of Medicine, Gunma University (Protocol number HS2020‐132).

## Supporting information


Figure S1

Figure S2

Figure S3

Tables S1‐S4
