# Supplementary figures and images for: Primary tracheobronchial necrosis after esophagectomy: A nationwide multicenter retrospective study in Japan
Source: Ann Gastroenterol Surg. 2022 Oct 8;7(2):236–46. doi: 10.1002/ags3.12625 (PMC10043765; doi:10.1002/ags3.12625)

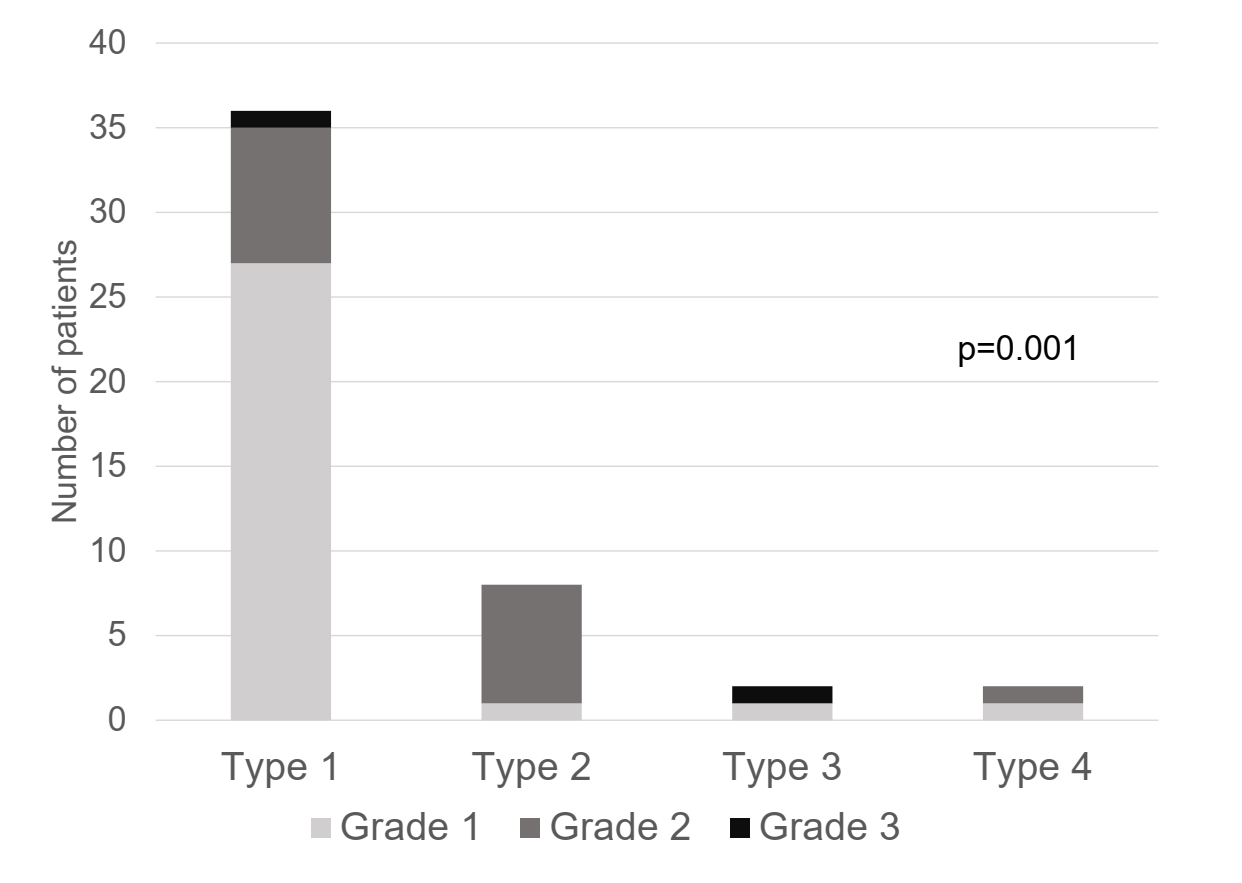

Supplement: Supplementary file 1 — Figure S1 [file AGS3-7-236-s001.tif]

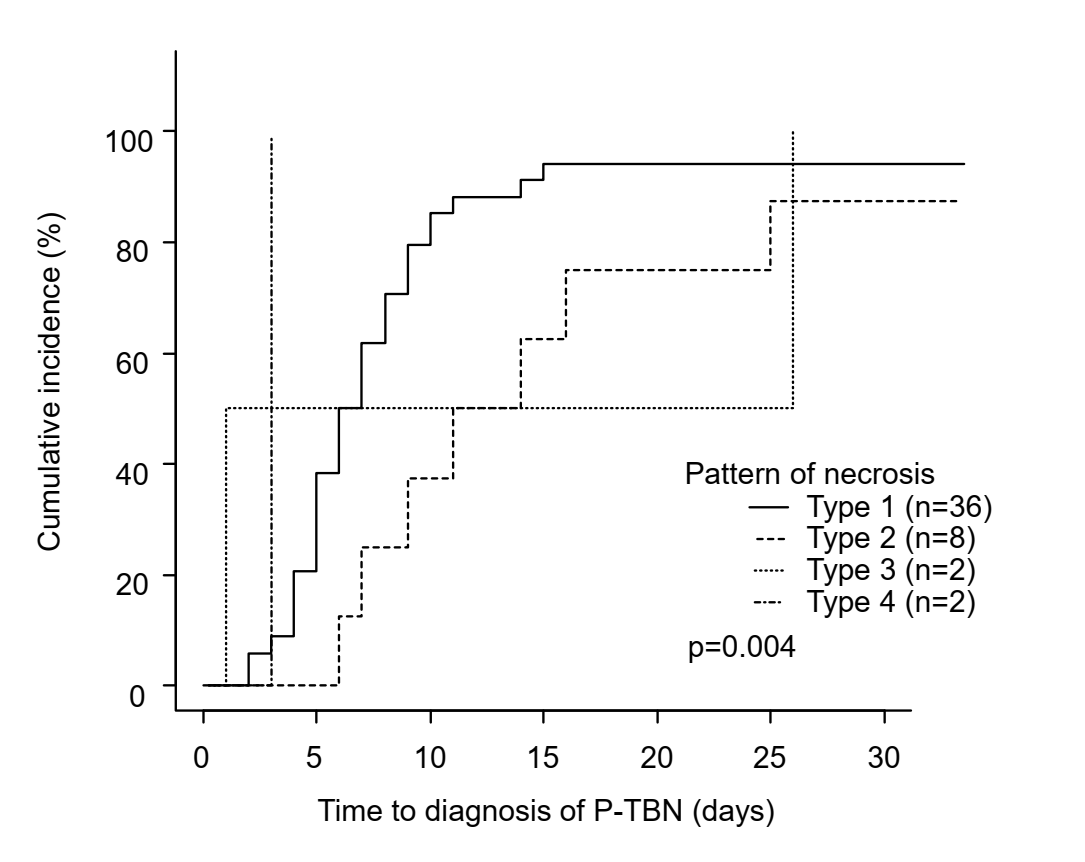

Supplement: Supplementary file 2 — Figure S2 [file AGS3-7-236-s002.tif]

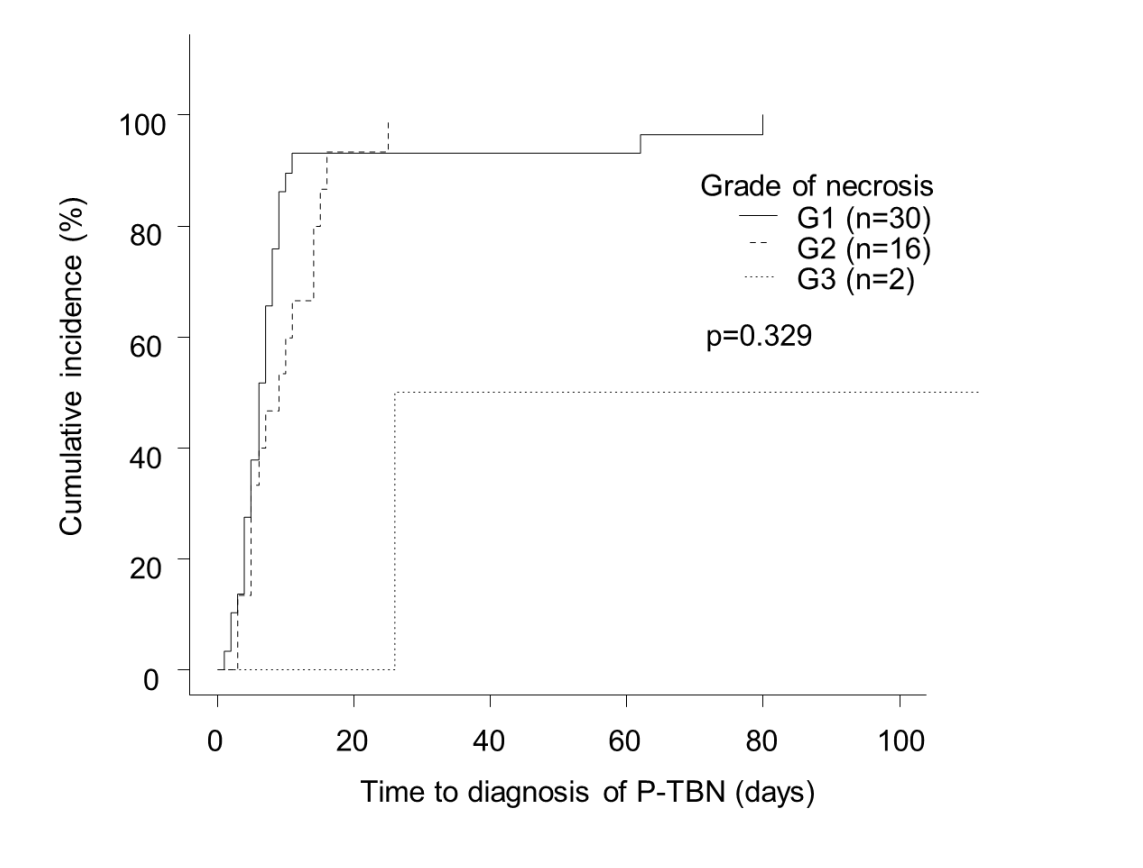

Supplement: Supplementary file 3 — Figure S3 [file AGS3-7-236-s004.tif]
