# Supplementary material for: Primary tracheobronchial necrosis after esophagectomy: A nationwide multicenter retrospective study in Japan
Source: Ann Gastroenterol Surg. 2022 Oct 8;7(2):236–46. doi: 10.1002/ags3.12625 (PMC10043765; doi:10.1002/ags3.12625)
Supplement: Supplementary file 4 — Tables S1‐S4 [file AGS3-7-236-s003.docx]

**Supplementary Table 1. Association between preservation of BA and P-TBN - all cases**

| **Characteristics** | **Type of BA preservation** | | |  |
| --- | --- | --- | --- | --- |
|  | Bilateral preserved | Lateral divided | Bilateral divided | p-value |
| *All cases ^§^(%) |  |  |  | <0.001 |
| P-TBN Absent | 2,893 (46.1) | 3,176 (50.6) | 203 (3.2) |  |
| Present | 42 (89.4) | 4 (8.5) | 1 (2.1) |  |
| PLCE ^§^(%) |  |  |  | 0.999 |
| P-TBN Absent | 1,617 (100) | 0 | 0 |  |
| Present | 33 (100) | 0 | 0 |  |
| ^†^TPLE ^§^(%) |  |  |  | 0.271 |
| P-TBN Absent | 73 (47.1) | 76 (49) | 6 (3.9) |  |
| Present | 6 (60.0) | 3 (30.0) | 1 (10.0) |  |
| ^‡^SE ^§^(%) |  |  |  | 0.098 |
| P-TBN Absent | 1,203 (26.7) | 3,100 (68.9) | 197 (4.4) |  |
| Present | 3 (75.0) | 1 (25.0) | 0 |  |

Abbreviations: BA, bronchial arteries; P-TBN, Primary tracheobronchial necrosis; PLCE, Pharyngo-laryngo-cervical esophagectomy; TPLE, Total pharyngo-laryngo-esophagectomy; SE, Subtotal esophagectomy.

*51 unknown cases were excluded, ^†^40 unknown cases were excluded, ^‡^11 unknown cases were excluded, ^§^row percentage

**Supplementary Table 2. Characteristics of patients undergoing PLCE - all cases**

| **Characteristics** | **All patients** | **P-TBN** | | |
| --- | --- | --- | --- | --- |
|  |  | Absent | Present | p-value |
|  | (n=1,650) | (n=1,617) | (n=33) |  |
| Type of tumor (%)r |  |  |  | 0.002 |
| Laryngeal | 85 (5.2) | 83 (5.1) | 2 (6.1) |  |
| Hypopharyngeal | 1,332 (80.7) | 1,313 (81.2) | 19 (57.6) |  |
| Esophageal | 216 (13.1) | 205 (12.7) | 11 (33.3) |  |
| Multiple primary | 3 (0.2) | 2 (0.1) | 1 (3.0) |  |
| Others | 14 (0.8) | 14 (0.9) | 0 |  |
| Preoperative treatment (%) |  |  |  | <0.001 |
| None | 1,108 (67.2) | 1,093 (67.6) | 15 (45.5) |  |
| Chemotherapy | 358 (21.7) | 354 (21.9) | 4 (12.1) |  |
| CRT or RT | 184 (11.2) | 170 (10.5) | 14 (42.4) |  |
| Unknown | 0 | 0 | 0 |  |
| Salvage surgery (%) |  |  |  | <0.001 |
| Negative | 1,369 (83) | 1,350 (83.5) | 19 (57.6) |  |
| Positive | 281 (17) | 267 (16.5) | 14 (42.4) |  |
| Unknown | 0 | 0 | 0 |  |
| Type of Reconstruction (%) |  |  |  | 0.077 |
| Jejunum: Free | 1,540 (93.3) | 1,510 (93.4) | 30 (90.9) |  |
| Skin flap | 17 (1.0) | 17 (1.1) | 0 |  |
| Musculocutaneous flap | 58 (3.5) | 58 (3.6) | 0 |  |
| Mixed | 35 (2.1) | 32 (2.0) | 3 (9.1) |  |

Abbreviations: P-TBN, Primary tracheobronchial necrosis; PLCE, Pharyngo-laryngo-cervical esophagectomy; CRT, chemoradiotherapy; RT, radiotherapy

**Supplementary Table 3. Characteristics of patients undergoing TPLE - all cases**

| **Characteristics** | **All patients** | **P-TBN** | | |
| --- | --- | --- | --- | --- |
|  |  | Absent | Present | p-value |
|  | (n=205) | (n=194) | (n=11) |  |
| Type of tumor (%) |  |  |  | 0.115 |
| Laryngeal | 2(0.5) | 2 (1.0) | 0 |  |
| Hypopharyngeal | 55 (26.7) | 54 (27.8) | 1 (9.1) |  |
| Esophageal | 111 (53.9) | 106 (54.6) | 5 (45.5) |  |
| Multiple primary | 37 (18.0) | 32 (16.5) | 5 (45.5) |  |
| Others | 0 | 0 | 0 |  |
| Preoperative treatment (%) |  |  |  | 0.314 |
| None | 122 (59.5) | 117 (60.3) | 5 (45.5) |  |
| Chemotherapy | 49 (23.9) | 44 (22.7) | 5 (45.5) |  |
| CRT or RT | 19 (9.3) | 18 (9.3) | 1 (9.1) |  |
| Unknown | 15 (7.3) | 15 (7.7) | 0 |  |
| Salvage surgery (%) |  |  |  | 0.632 |
| Negative | 156 (76.1) | 146 (75.3) | 10 (90.9) |  |
| Positive | 34 (16.6) | 33 (17.0) | 1 (9.1) |  |
| Unknown | 15 (7.3) | 15 (7.7) | 0 |  |
| Type of Reconstruction (%) |  |  |  | 0.831 |
| Whole stomach | 15 (7.3) | 13 (6.7) | 2 (18.2) |  |
| Gastric tube | 114 (55.6) | 108 (55.7) | 6 (54.5) |  |
| Jejunum (pedicled) | 6 (2.9) | 6 (3.1) | 0 |  |
| Jejunum (free) | 11 (5.4) | 11 (5.7) | 0 |  |
| Colon (pedicled) | 5 (2.4) | 5 (2.6) | 0 |  |
| Colon (free) | 1 (0.5) | 1 (0.5) | 0 |  |
| Skin flap | 2 (1.0) | 2 (1.0) | 0 |  |
| Musculocutaneous flap | 5 (2.4) | 5 (2.6) | 0 |  |
| Mixed | 46 (22.4) | 43 (22.2) | 3 (27.3) |  |
| Reconstruction routes (%) |  |  |  | 0.136 |
| Antethoracic (subcutaneous) | 8 (3.9) | 8 (4.1) | 0 |  |
| Retrosternal | 12 (5.9) | 10 (5.2) | 2 (18.2) |  |
| Posterior mediastinal | 149 (72.7) | 140 (72.2) | 9 (81.8) |  |
| Unknown | 36 (17.6) | 36 (18.6) | 0 |  |

Abbreviations: P-TBN, Primary tracheobronchial necrosis; TPLE, Total pharyngo-laryngo-esophagectomy; CRT, chemoradiotherapy; RT, radiotherapy

**Supplementary Table 4. Characteristics of patients undergoing SE - all cases**

| **Characteristics** | **All patients** | **P-TBN** | | |
| --- | --- | --- | --- | --- |
|  |  | Absent | Present | p-value |
|  | (n=4,515) | (n=4,511) | (n=4) |  |
| Type of tumor (%) |  |  |  | 0.999 |
| Laryngeal | 0 | 0 | 0 |  |
| Hypopharyngeal | 0 | 0 | 0 |  |
| Esophageal | 4,515 | 4,511 | 4 |  |
| Multiple primary | 0 | 0 | 0 |  |
| Others | 0 | 0 | 0 |  |
| Preoperative treatment (%) |  |  |  | 0.490 |
| None | 2,214 (49.0) | 2,213 (49.1) | 1 (25.0) |  |
| Chemotherapy | 1,539 (34.1) | 1,537 (34.1) | 2 (50.0) |  |
| CRT or RT | 476 (10.5) | 475 (10.5) | 1 (25.0) |  |
| Unknown | 286 (6.3) | 286 (100) | 0 |  |
| Salvage surgery (%) |  |  |  | 0.632 |
| Negative | 4,054 (89.8) | 4,050 (89.8) | 4 (100) |  |
| Positive | 175 (3.9) | 175 (3.9) | 0 |  |
| Unknown | 286 (6.3) | 286 (6.3) | 0 |  |
| Type of Reconstruction (%) |  |  |  | 0.999 |
| Whole stomach | 9 (0.2) | 9 (0.2) | 0 |  |
| Gastric tube | 4,265 (94.5) | 4,261 (94.5) | 4 (100) |  |
| Jejunum (pedicled) | 51 (1.1) | 51 (1.1) | 0 |  |
| Jejunum (free) | 25 (0.6) | 25 (0.6) | 0 |  |
| Colon (pedicled) | 150 (3.3) | 150 (3.3) | 0 |  |
| Colon (free) | 7 (0.2) | 7 (0.2) | 0 |  |
| Skin flap | 0 | 0 | 0 |  |
| Musculocutaneous flap | 0 | 0 | 0 |  |
| Mixed | 8 (0.2) | 8 (0.2) | 0 |  |
| Reconstruction routes (%) |  |  |  | 0.283 |
| Antethoracic (subcutaneous) | 416 (9.2) | 416 (9.2) | 0 |  |
| Retrosternal | 1,703 (37.7) | 1,703 (37.8) | 0 |  |
| Posterior mediastinal | 2,102 (46.6) | 2,098 (46.5) | 4 (100) |  |
| Unknown | 294 (6.5) | 294 (6.5) | 0 |  |

Abbreviations: P-TBN, Primary tracheobronchial necrosis; SE, subtotal esophagectomy; CRT, chemoradiotherapy; RT, radiotherapy
